# Trotabresib (CC-90010) in combination with adjuvant temozolomide or concomitant temozolomide plus radiotherapy in patients with newly diagnosed glioblastoma

**DOI:** 10.1093/noajnl/vdac146

**Published:** 2022-10-28

**Authors:** Maria Vieito, Matteo Simonelli, Filip de Vos, Victor Moreno, Marjolein Geurts, Elena Lorenzi, Marina Macchini, Martin J van den Bent, Gianluca Del Conte, Maja de Jonge, Maria Cruz Martín-Soberón, Barbara Amoroso, Tania Sanchez-Perez, Marlene Zuraek, Bishoy Hanna, Ida Aronchik, Ellen Filvaroff, Henry Chang, Cristina Mendez, Marina Arias Parro, Xin Wei, Zariana Nikolova, Juan Manuel Sepulveda

**Affiliations:** Vall d’Hebron Institute of Oncology (VHIO), Barcelona, Spain; Universidad Autonoma de Barcelona, Barcelona, Spain; Department of Biomedical Sciences, Humanitas University, Milan, Italy; IRCCS Humanitas Research Hospital, Milan, Italy; Department of Medical Oncology, University Medical Center Utrecht, Utrecht University, Utrecht, the Netherlands; START Madrid-FJD, Hospital Universitario Fundación Jiménez Díaz, Madrid, Spain; Erasmus MC Cancer Institute, Rotterdam, the Netherlands; IRCCS Humanitas Research Hospital, Milan, Italy; Department of Oncology, IRCCS San Raffaele Scientific Institute, Milan, Italy; Erasmus MC Cancer Institute, Rotterdam, the Netherlands; Department of Oncology, IRCCS San Raffaele Scientific Institute, Milan, Italy; Erasmus MC Cancer Institute, Rotterdam, the Netherlands; Neuro-Oncology Unit, Department of Medical Oncology, Hospital Universitario 12 de Octubre, Madrid, Spain; Centre for Innovation and Translational Research Europe, A Bristol Myers Squibb Company, Seville, Spain; Centre for Innovation and Translational Research Europe, A Bristol Myers Squibb Company, Seville, Spain; Bristol Myers Squibb, San Francisco, CA, USA; Bristol Myers Squibb, Princeton, NJ, USA; Bristol Myers Squibb, San Francisco, CA, USA; Bristol Myers Squibb, San Francisco, CA, USA; Bristol Myers Squibb, San Francisco, CA, USA; Centre for Innovation and Translational Research Europe, A Bristol Myers Squibb Company, Seville, Spain; Bristol Myers Squibb, Boudry, Switzerland; Bristol Myers Squibb, Princeton, NJ, USA; Centre for Innovation and Translational Research Europe, A Bristol Myers Squibb Company, Seville, Spain; Hospital Universitario 12 de Octubre, Madrid, Spain

**Keywords:** trotabresib, BET inhibitors, glioblastoma, temozolomide, pharmacokinetics

## Abstract

**Background:**

Standard-of-care treatment for newly diagnosed glioblastoma (ndGBM), consisting of surgery followed by radiotherapy (RT) and temozolomide (TMZ), has improved outcomes compared with RT alone; however, prognosis remains poor. Trotabresib, a novel bromodomain and extraterminal inhibitor, has demonstrated antitumor activity in patients with high-grade gliomas.

**Methods:**

In this phase Ib, dose-escalation study (NCT04324840), we investigated trotabresib 15, 30, and 45 mg combined with TMZ in the adjuvant setting and trotabresib 15 and 30 mg combined with TMZ+RT in the concomitant setting in patients with ndGBM. Primary endpoints were to determine safety, tolerability, maximum tolerated dose, and/or recommended phase II dose (RP2D) of trotabresib. Secondary endpoints were assessment of preliminary efficacy and pharmacokinetics. Pharmacodynamics were investigated as an exploratory endpoint.

**Results:**

The adjuvant and concomitant cohorts enrolled 18 and 14 patients, respectively. Trotabresib in combination with TMZ or TMZ+RT was well tolerated; most treatment-related adverse events were mild or moderate. Trotabresib pharmacokinetics and pharmacodynamics in both settings were consistent with previous data for trotabresib monotherapy. The RP2D of trotabresib was selected as 30 mg 4 days on/24 days off in both settings. At last follow-up, 5 (28%) and 6 (43%) patients remain on treatment in the adjuvant and concomitant settings, respectively, with 1 patient in the adjuvant cohort achieving complete response.

**Conclusions:**

Trotabresib combined with TMZ in the adjuvant setting and with TMZ+RT in the concomitant setting was safe and well tolerated in patients with ndGBM, with encouraging treatment durations. Trotabresib 30 mg was established as the RP2D in both settings.

**Key Points**

- Trotabresib combined with adjuvant TMZ or concomitant TMZ + RT was well tolerated.
- TMZ and RT did not impact trotabresib pharmacokinetics or pharmacodynamics.
- Preliminary efficacy with trotabresib + TMZ ± RT appeared encouraging.

**Importance of the Study**

Standard-of-care (SOC) therapy for newly diagnosed glioblastoma (ndGBM) is maximal surgical resection followed by concomitant radiotherapy and temozolomide and adjuvant temozolomide; however, prognosis remains poor. Our study investigates trotabresib, a novel, oral, reversible bromodomain and extraterminal inhibitor that penetrates the blood–brain barrier, combined with adjuvant temozolomide and concomitant temozolomide plus radiotherapy in patients with ndGBM. Trotabresib was well tolerated in both settings. Median duration of treatment was 33 and 34 weeks in the adjuvant and concomitant settings, respectively. Six-month progression-free survival rates were 57.8% and 69.2% in the adjuvant and concomitant settings, respectively. The recommended phase II dose of trotabresib was established as 30 mg. Based on these results, patient enrollment has started for the randomized phase II dose expansion, which will compare concomitant trotabresib 30 mg plus temozolomide and radiotherapy followed by adjuvant trotabresib 30 mg plus temozolomide then maintenance with trotabresib 45 mg monotherapy versus SOC.

Glioblastoma, the most common and aggressive primary brain tumor in adults, is challenging to treat and remains incurable despite extensive research.^1^ Standard-of-care (SOC) therapy for newly diagnosed glioblastoma, consisting of maximal safe surgical resection followed by radiotherapy (RT) and concomitant temozolomide (TMZ), followed by adjuvant TMZ, was established over 15 years ago.^2^ TMZ plus RT significantly improved survival compared with RT alone, with a hazard ratio for overall survival (OS) of 0.6 (95% CI: 0.5–0.7; *P* < .0001).^3,4^ However, the poor prognosis for patients with glioblastoma is highlighted by the low 6-month progression-free survival (PFS) rate of 53.9% and 5-year OS rate of 9.8% in patients receiving TMZ plus RT.^3,4^ A key contributor to poor outcomes is the high rate of recurrence due to extensive tumor infiltration of surrounding brain tissue and the difficulty of achieving complete surgical resection.^1,5,6^ Clinical trials have found many otherwise promising therapies to have minimal antitumor activity, with poor blood–brain barrier (BBB) penetration thought to be a key contributing factor.^7,8^ Given the unmet medical needs of patients with glioblastoma, new therapies with novel mechanisms of action and the ability to cross the BBB are urgently needed to improve patient outcomes.

The bromodomain and extraterminal (BET) family of proteins, comprising BRD2, BRD3, BRD4, and BRDT, are epigenetic readers that regulate the expression of key genes involved in oncogenesis, apoptosis, and cell cycle progression.^9,10^ BET proteins have been found to be overexpressed in a range of tumor types, including glioblastoma, and elevated levels of BRD2 and BRD4 are associated with poor prognosis in patients with glioblastoma.^10–14^ BET inhibitors have been shown to regulate expression of key genes associated with glioblastoma proliferation, including *MYC*, *CDKN1A*, and *BCL2L1*, and to decrease glioblastoma cell growth in vitro and in patient-derived xenograft (PDX) models.^10,15,16^ BET proteins are therefore relevant therapeutic targets in cancer. Inhibition of BET proteins has demonstrated antitumor activity in clinical trials across a range of advanced cancers,^17–19^ supporting investigation of BET inhibitors for the treatment of glioblastoma. However, although preclinical studies of various BET inhibitors have shown antitumor effects in glioblastoma models (reviewed in Yang et al^20^), clinical investigation has been limited, with a dose-finding study of OTX015 in patients with glioblastoma being discontinued due to lack of clinical activity after failing to meet its primary endpoint of 6-month PFS,^21^ suggestive of poor BBB penetration in humans and/or nonoptimal dosing.

Trotabresib (CC-90010; BMS-986378) is a novel, oral, reversible, small-molecule inhibitor of BET proteins. In the first-in-human CC-90010-ST-001 study, trotabresib demonstrated encouraging antitumor activity across a range of doses and dosing schedules in patients with advanced malignancies, including durable responses in patients with high-grade gliomas (V. Moreno, unpublished manuscript). In a “window-of-opportunity” phase I study, CC-90010-GBM-001, patients with recurrent high-grade glioma who were scheduled for salvage resection received trotabresib 30 mg/day on days 1–4 before surgery, followed by maintenance trotabresib monotherapy 45 mg administered on days 1–4 of each 28-day cycle (4 days on/24 days off) after surgery, with the aim of evaluating trotabresib BBB penetration.^22^ The study found that trotabresib reached detectable concentrations in resected contrast-enhancing and non-enhancing brain tumor tissue, with a brain tumor tissue:plasma ratio of 0.84. Trotabresib was well tolerated, and 2 patients remained on treatment in cycles 19 and 23, with durable stable disease (SD) at the time of last follow-up.^22^

Preclinical studies have demonstrated that trotabresib has antitumor activity in glioblastoma models as a single agent and enhances the antiproliferative effects of TMZ in glioblastoma PDX models (Supplementary Figure S1). TMZ inhibits DNA replication through addition of alkyl groups to the O^6^ position of guanines. Importantly, expression of O^6^-methylguanine-DNA methyltransferase (MGMT), an enzyme that removes alkyl groups from DNA, is associated with decreased benefit from TMZ therapy, and methylation of the *MGMT* promoter is associated with improved OS in patients treated with TMZ.^23–28^ Preclinical studies indicate that trotabresib downregulates *MGMT* expression in a dose-dependent manner (Supplementary Figure S2), providing further support to the combination of trotabresib and TMZ. Trotabresib has demonstrated antiproliferative activity as a single agent in glioblastoma PDX models, regardless of *MGMT* promoter methylation status; these effects may be exerted by modulating the expression of other genes, such as *MYC*. *MYC* is often overexpressed in glioblastoma^29,30^ and has been shown to be downregulated in vitro by trotabresib (Bristol Myers Squibb; data on file) and the BET inhibitor, JQ1.^15^

A rationale for combining BET inhibition with RT has been provided in preclinical studies. In rhabdomyosarcoma cells, OTX015 demonstrated antitumor activity as a single agent and, by downregulating drivers of cell proliferation and resistance, potentiated the effects of ionizing radiation.^31^ In breast cancer cells, iBET-762 upregulated expression of the HER2-regulated gene, *TUBB3*, and increased sensitivity to vinorelbine in vitro and in PDX models. In a mouse model of breast cancer brain metastases, longer survival was observed in mice treated with iBET-762 combined with vinorelbine plus radiation than in mice treated with radiation alone, or iBET-762 or vinorelbine in combination with radiation.^32^

CC-90010-GBM-002 (NCT04324840) is a phase Ib/II study in adult patients with newly diagnosed grade IV glioblastoma who have undergone complete or partial tumor resection. The study is investigating trotabresib in combination with TMZ in the adjuvant setting followed by maintenance trotabresib monotherapy, and trotabresib combined with TMZ and RT in the concomitant setting followed by adjuvant trotabresib plus TMZ, followed by maintenance trotabresib monotherapy. Here, we present safety, pharmacokinetics, pharmacodynamics, and efficacy data for patients enrolled in the adjuvant and concomitant dose-escalation cohorts (part A).

## Materials and Methods

### Study Design

**Figure 1.**
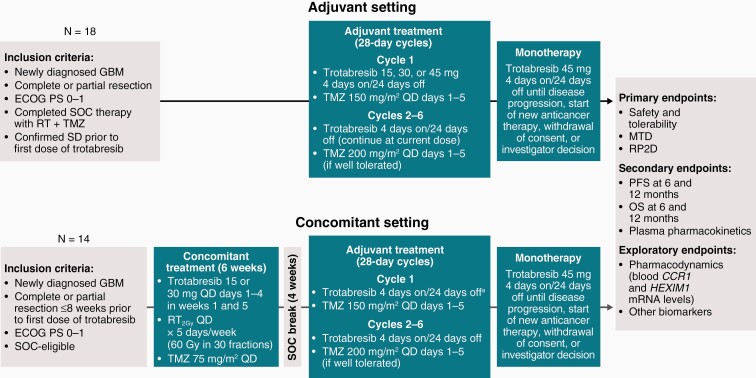
CC-90010-GBM-002 study design. ^a^Administered at the highest dose level that was well tolerated by at least 1 cohort in the adjuvant dose escalation. Abbreviations: ECOG PS, Eastern Cooperative Oncology Group performance status; GBM, glioblastoma; MTD, maximum tolerated dose; OS, overall survival; PFS, progression-free survival; QD, daily; RP2D, recommended phase II dose; RT, radiotherapy; RT_2Gy_, radiotherapy at 2 Gray per fraction; SD, stable disease; SOC, standard-of-care; TMZ, temozolomide.

CC-90010-GBM-002 is a multicenter, open-label, phase Ib/II, dose-finding study in adult patients with newly diagnosed glioblastoma. The phase Ib, dose-escalation part of this study explored escalating oral doses of trotabresib in combination with TMZ in the adjuvant setting and with TMZ plus RT in the concomitant setting. In the adjuvant setting, patients received 6 cycles of trotabresib administered at doses of 15, 30, and 45 mg 4 days on/24 days off in combination with TMZ administered per label,^33,34^ followed by trotabresib 45 mg 4 days on/24 days off as maintenance therapy. After two dose-escalation levels were assessed as safe and tolerable in the adjuvant therapy cohort, the concomitant therapy dose-escalation portion was initiated, in which patients received 6 weeks of treatment with trotabresib 15 or 30 mg, administered on days 1–4 in weeks 1 and 5, in combination with concomitant TMZ and RT administered per label.^33,34^ After completion of the concomitant treatment stage, patients had a 4-week treatment break then received adjuvant trotabresib administered 4 days on/24 days off at the highest dose level that was well tolerated by at least 1 cohort in the adjuvant dose escalation, plus TMZ administered per label, followed by maintenance trotabresib 45 mg administered 4 days on/24 days off. Dose-escalation decisions were made by the safety review committee, based on all available safety, pharmacokinetics, pharmacodynamics, and preliminary efficacy information, and a calculation of risk assessment using a Bayesian logistic regression model. The study design is shown in Figure 1.

### Patient Selection

Eligible patients were ≥18 years of age, had newly diagnosed, histologically confirmed World Health Organization grade IV glioblastoma (2016 classification) and had undergone complete or partial tumor resection, and an Eastern Cooperative Oncology Group performance status (ECOG PS) of 0 or 1. Patients must not have had a requirement for ongoing treatment with therapeutic or prophylactic anticoagulants.

For enrollment in the adjuvant cohort, patients must have recently completed a course of concomitant TMZ plus standard or hypofractionated RT, with an MRI documenting SD prior to the first dose of trotabresib. For enrollment in the concomitant cohort, patients must have been eligible for treatment with TMZ plus RT and have undergone complete or partial tumor resection ≤8 weeks, but preferably ≤6 weeks, prior to the first dose of trotabresib.

Key exclusion criteria were receipt of prior chemotherapy or other antitumor treatment for glioblastoma (either approved or investigational) except for surgery (and for patients in the adjuvant therapy cohort, mandatory concomitant TMZ plus RT). Patients were also excluded if they had any known metastatic extracranial or leptomeningeal disease.

Additional exclusion criteria included mild or asymptomatic SARS-CoV-2 infection within 10 days or severe/critical SARS-CoV-2 infection within 20 days prior to cycle 1, day 1 (C1D1). Acute symptoms must have resolved, and based on investigator assessment in consultation with the medical monitor, there must be no sequelae that would place the patient at increased risk while receiving study treatment. Patients receiving previous SARS-CoV-2 vaccine within 14 days prior to C1D1 were excluded; for vaccines requiring more than 1 dose, the full series (e.g., both doses of a 2-dose series) should have been completed prior to C1D1 when feasible and when a delay in C1D1 would not put the patient at risk. The administration of a live SARS-CoV-2 vaccine was prohibited up to 14 days prior to the initiation of study treatment.

A full description of inclusion and exclusion criteria is provided in Supplementary Table S1.

All patients provided written informed consent, and the study was conducted in compliance with the International Council on Harmonisation of Technical Requirements for Registration of Pharmaceuticals for Human Use, Good Clinical Practice, and general ethical principles outlined in the Declaration of Helsinki.

### Endpoints and Assessments

The primary endpoints of the study were to determine the safety and tolerability, maximum tolerated dose, and/or recommended phase II dose (RP2D) of trotabresib in combination with TMZ as adjuvant therapy, and with TMZ and RT as concomitant therapy. Secondary endpoints included assessment of preliminary efficacy in terms of PFS (defined as the time from randomization to the date of first documented tumor progression or death prior to imaging showing progression) and OS rates at 6 and 12 months and antitumor response, as well as the plasma pharmacokinetics of trotabresib. Trotabresib pharmacodynamics was an exploratory endpoint.

Adverse events (AEs) were classified using the Medical Dictionary for Regulatory Activities v18.1 or higher system organ class and preferred term. Severity of AEs was graded based on the patient’s symptoms according to the National Cancer Institute Common Terminology Criteria for Adverse Events v5.0.^35^ Dose-limiting toxicities (DLTs) were defined as any of the toxicities described in Supplementary Table S2 occurring within the DLT assessment period (cycle 1 [28 days]), unless the event could clearly be determined to be unrelated to trotabresib. Screening MRI scans were performed after surgical resection and within 28 days (+3 days) prior to initiation of study treatment. Subsequent tumor assessments by MRI or CT were performed at week 10 during the concomitant treatment period and at the end of every other treatment cycle from cycle 2 onwards during the adjuvant and trotabresib monotherapy treatment periods. Best overall response (BOR) was evaluated as complete response (CR), partial response (PR), SD, progressive disease (PD), or not evaluable, according to Response Assessment in Neuro-Oncology criteria for high-grade glioma.^36^ Plasma samples were collected during the first treatment cycle for investigation of trotabresib pharmacokinetics and pharmacodynamics. Plasma pharmacokinetic parameters assessed included peak plasma concentration (*C*_max_), time to peak plasma concentration (*t*_max_), area under the plasma concentration–time curve (AUC), and terminal half-life (*t*_½_). Pharmacodynamic markers assessed included blood levels of *CCR1* and *HEXIM1* mRNA, which are established markers of BET inhibitor target engagement.^17,22,37,38^

Baseline isocitrate dehydrogenase (*IDH*) mutation status and *MGMT* promoter methylation status were determined in surgically resected brain tumor tissue using recently obtained tissue from surgery and assessed per methodologies adopted at each institution.

The sample size was not based on strict statistical power calculations. Safety, efficacy, and pharmacodynamic analyses were summarized using descriptive statistics. Plasma pharmacokinetic parameters were calculated by the noncompartmental analysis method from plasma concentration–time profiles.

## Results

### Patients and Treatment

Patient enrollment for part A was completed on July 5, 2021. Eighteen patients were enrolled in the adjuvant cohort and received trotabresib 15 mg (*n* = 5), 30 mg (*n* = 6), or 45 mg (*n* = 7) 4 days on/24 days off plus TMZ. Fourteen patients were enrolled in the concomitant cohort and received trotabresib 15 mg (*n* = 6) or 30 mg (*n* = 8) 4 days on/24 days off plus TMZ and RT.

At the time of preparing this report (May 11, 2022), 5 (28%) patients in the adjuvant cohort and 6 (43%) patients in the concomitant cohort remained on treatment. In the adjuvant cohort, 8 (44%) patients discontinued due to PD, 3 (17%) patients discontinued due to AEs, 1 (6%) patient discontinued due to investigator decision, and 1 (6%) patient discontinued due to radionecrosis. In the concomitant cohort, 4 (29%) patients discontinued due to PD, 3 (21%) patients withdrew consent, and 1 (7%) patient discontinued due to an AE. Among patients who withdrew consent, 1 withdrew due to the perceived burden of multiple grade 1/2 AEs caused by study treatments, 1 lived far from the institution and family members were unable to assist with travel and domestic matters, and 1 preferred to proceed with off-protocol SOC therapy. Following treatment discontinuation, 3 (17%) patients and 1 (7%) patient in the adjuvant and concomitant cohorts, respectively, died following disease progression.

Baseline demographics and characteristics are described in Table 1. Median age was 53 and 56 years in the adjuvant and concomitant cohorts, respectively. Twelve (67%) and 8 (57%) patients were male in the adjuvant and concomitant cohorts, respectively. Fourteen (78%) and 6 (43%) patients had an ECOG PS of 0 in the adjuvant and concomitant cohorts, respectively.

**Table 1.** Patient demographics and characteristics

Patient characteristic	Adjuvant overall (*N* = 18)	Concomitant overall (*N* = 14)
Median age (range), years	53 (31–70)	56 (29–71)
Sex, *n* (%)		
Male	12 (67)	8 (57)
Female	6 (33)	6 (43)
ECOG PS, *n* (%)		
0	14 (78)	6 (43)
1	4 (22)	8 (57)
Type of resection, *n* (%)		
Complete	10 (56)	9 (64)
Partial	8 (44)	5 (36)
*MGMT* promoter methylation status, *n* (%)		
Methylated	6 (33)	7 (50)
Unmethylated	10 (56)	3 (21)
Not reported	2 (11)	4 (29)
*IDH* mutation status, *n* (%)		
Wild type	17 (94)	11 (79)
Mutant	1 (6)	2 (14)
Not reported	0	1 (7)

Abbreviation: ECOG PS, Eastern Cooperative Oncology Group performance status.

### Safety

**Figure 2.**
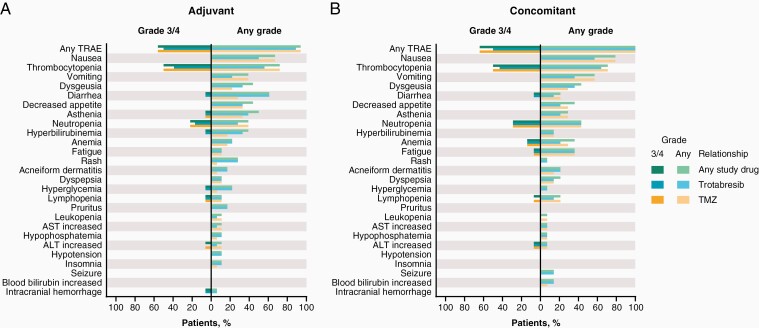
TRAEs reported in ≥2 patients at any grade or in ≥1 patient at grade 3/4 severity in the (A) adjuvant and (B) concomitant cohorts by relationship to study drug. Abbreviations: ALT, alanine aminotransferase; AST, aspartate aminotransferase; TMZ, temozolomide; TRAE, treatment-related adverse event.

At the February 20, 2022, data cutoff, any-grade treatment-related AEs (TRAEs) were reported in 17 (94%) and 14 (100%) patients in the overall adjuvant and concomitant cohorts, respectively. Grade 3/4 TRAEs were reported in 10 (56%) and 9 (64%) patients in the adjuvant and concomitant cohorts, respectively (Figure 2 and Supplementary Table S3).

In the overall adjuvant cohort, thrombocytopenia was the most common any-grade and grade 3/4 TRAE related to any study drug, reported in 13 (72%) and 9 (50%) patients, respectively (Figure 2A and Supplementary Table S3). Thrombocytopenia was attributed to trotabresib in 10 (56%) and 7 (39%) patients at any grade and grade 3/4, respectively. Gastrointestinal TRAEs related to any study drug occurred frequently (reported in 14 [78%] patients); however, grade 3/4 gastrointestinal  TRAEs were reported in only 1 (6%) patient (diarrhea). Rash and other skin-related TRAEs were reported in 9 (50%) patients, all grade 1 or 2, and were attributed to both trotabresib and TMZ in 2 (11%) patients and to trotabresib only in 7 (39%) patients.

In the overall concomitant cohort, nausea was the most common any-grade TRAE related to any study drug, reported in 11 (79%) patients, all grade 1 or 2, and was attributed to trotabresib in 8 (57%) patients (Figure 2B and Supplementary Table S3). Any-grade thrombocytopenia was reported in 10 (71%) patients and was attributed to trotabresib in 9 (64%) patients. Thrombocytopenia was the most common grade 3/4 TRAE, reported in 7 (50%) patients, and was attributed to trotabresib in 6 (43%) patients. Gastrointestinal  TRAEs related to any study drug occurred in 13 (93%) of patients at any grade, with a grade 3/4 TRAE reported in 1 (7%) patient (diarrhea). Rash and other skin-related TRAEs were reported in 7 (50%) patients, all grade 1 or 2, and were attributed to both trotabresib and TMZ in 3 (21%) patients and to trotabresib only in 4 (29%) patients.

The incidence of TRAEs attributed to TMZ is shown in Supplementary Table S3, and the overall incidence of TRAEs at each dose level in the adjuvant and concomitant cohorts is shown in Supplementary Table S4.

Serious TRAEs were reported in 3 (17%) patients and 1 (7%) patient in the adjuvant and concomitant cohorts, respectively. In the adjuvant cohort at the trotabresib 30 mg 4 days on/24 days off dose level, serious neutropenia and thrombocytopenia, attributed to both trotabresib and TMZ, were reported in 1 patient, and serious intracranial hemorrhage, attributed to trotabresib, was reported in 1 patient. Serious diarrhea, attributed to trotabresib, was reported in 1 patient who received trotabresib 45 mg 4 days on/24 days off in the adjuvant cohort. Serious bone marrow failure, attributed to both trotabresib and TMZ, was reported in 1 patient in the concomitant cohort who was treated at the trotabresib 30 mg 4 days on/24 days off dose level. There were no treatment-related deaths.

In the adjuvant cohort, 2 (11%) patients permanently discontinued trotabresib and TMZ due to treatment-emergent AEs (TEAEs) considered unrelated to treatment in 1 patient at the 45-mg dose level and related to both trotabresib and TMZ in 1 patient at the 30-mg dose level. No TEAEs leading to discontinuation of either study drug were reported in the concomitant cohort (Supplementary Table S5). One patient in the 30-mg adjuvant cohort discontinued treatment due to a DLT of thrombocytopenia. Details of treatment duration and relative dose intensities for trotabresib and TMZ are shown in Supplementary Table S6. The RP2D of trotabresib was established as 30 mg in the adjuvant and concomitant settings.

### Pharmacokinetics

**Figure 3.**
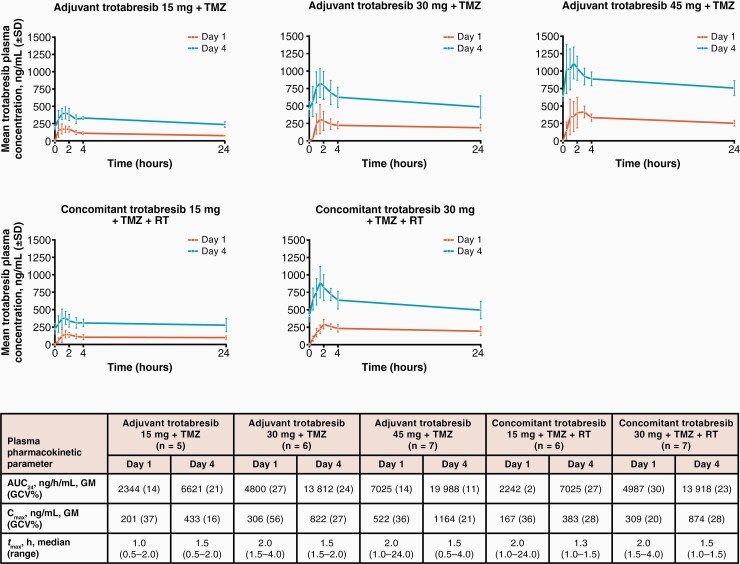
Trotabresib plasma pharmacokinetics. Abbreviations: AUC_24_, area under the trotabresib concentration–time curve from 0 to 24 hours; C_max_, peak trotabresib concentration; GCV, geometric coefficient of variation; GM, geometric mean; RT, radiotherapy; SD, standard deviation; t_max_, time to peak trotabresib concentration; TMZ, temozolomide.

Preliminary trotabresib plasma pharmacokinetic data were available for all 18 patients in the adjuvant setting and for 13 of 14 patients in the concomitant setting; 1 patient in the concomitant trotabresib 30-mg cohort did not have samples available for pharmacokinetics analysis. Increases in trotabresib exposure were proportional to dose in both treatment settings. Trotabresib *t*_max_ on day 4 was 0.5–4.0 h and mean *t*_½_ was 74.2 h (standard deviation ± 27.2; Figure 3). Trotabresib plasma pharmacokinetics at the RP2D of 30 mg were similar across the adjuvant and concomitant settings, and were consistent with the pharmacokinetics of trotabresib monotherapy,^17,22,37^ suggesting that trotabresib plasma pharmacokinetics were not affected by TMZ or RT.

### Pharmacodynamics

**Figure 4.**
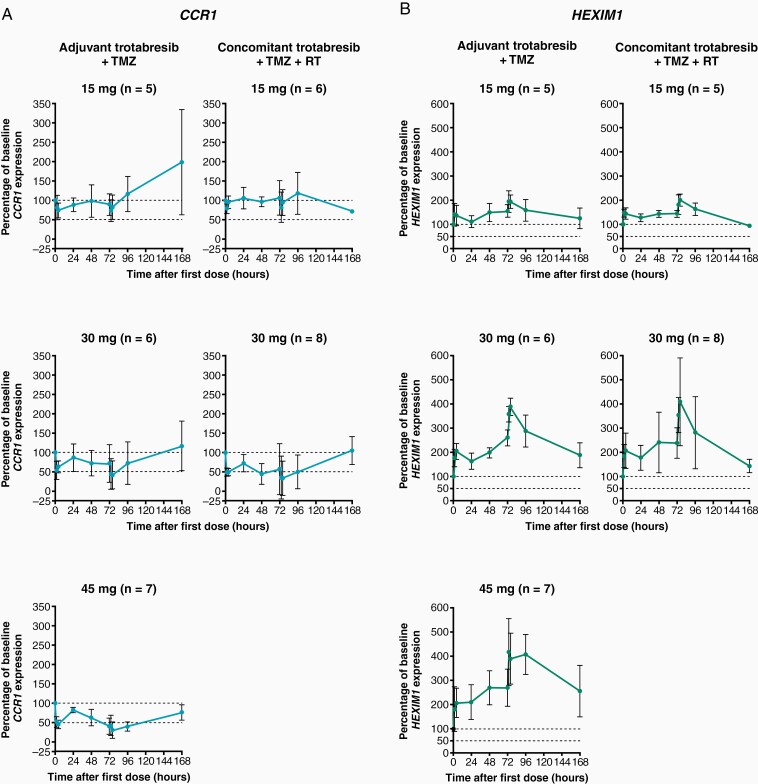
Trotabresib pharmacodynamics: modulation of (A) *CCR1* and (B) *HEXIM1* mRNA in blood. Abbreviations: RT, radiotherapy; TMZ, temozolomide.

In patients receiving trotabresib at the RP2D of 30 mg in the adjuvant and concomitant settings, blood pharmacodynamics were consistent with those observed in clinical trials investigating trotabresib monotherapy.^17,22,37^ On day 4, expression of blood *CCR1* mRNA at 2–4 h post-dose was ≥50% lower than at baseline in both the adjuvant and concomitant settings. Similar *CCR1* modulation was observed in patients treated with trotabresib 45 mg in the adjuvant cohort, but no decrease in *CCR1* expression was observed in patients treated with trotabresib 15 mg in either the adjuvant or concomitant cohorts. At the RP2D, *CCR1* mRNA expression returned to baseline levels by 168 h post-dose in cycle 1 (Figure 4A). At the RP2D in the adjuvant and concomitant cohorts, a 2.5- to 4.0-fold increase in blood *HEXIM1* mRNA expression was observed on days 3 and 4; *HEXIM1* mRNA expression remained above baseline until 168 h post-dose in cycle 1. *HEXIM1* modulation increased with trotabresib dose in both cohorts (Figure 4B) and showed an association with time-matched trotabresib plasma concentrations in an exposure–response analysis using pooled data from the current study and 2 other trotabresib studies, CC-90010-GBM-001 and CC-90010-ST-001 (Supplementary Figure S3).

### Efficacy

At the February 20, 2022, data cutoff, median duration of treatment was 33 weeks (range: 4–72) in the adjuvant cohort and 34 weeks (range: 8–54) in the concomitant cohort. Median PFS was 7.6 months (95% CI: 3.9–not estimable [NE]) in the adjuvant cohort and NE (95% CI: 5.2 months–NE) in the concomitant cohort. PFS rate at 6 months was 57.8% (95% CI: 31.1–77.3) in the adjuvant cohort and 69.2% (95% CI: 37.3–87.2) in the concomitant cohort (Supplementary Table S7). BOR for patients in the adjuvant and concomitant cohorts is shown in Supplementary Table S8.

**Figure 5.**
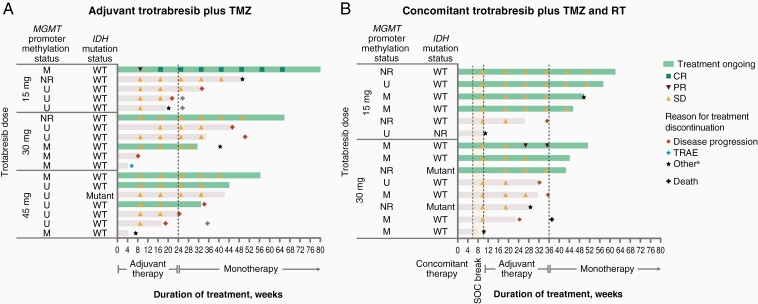
Treatment duration and response in the (A) adjuvant cohort and (B) concomitant cohort as of May 11, 2022. ^a^Includes withdrawal of consent and discontinuation due to TEAE or investigator decision. Abbreviations: CR, complete response; M, methylated; NR, not reported; PR, partial response; RT, radiotherapy; SD, stable disease; SOC, standard-of-care; TEAE, treatment-emergent adverse event; TMZ, temozolomide; TRAE, treatment-related adverse event; U, unmethylated; WT, wild type.

At the time of preparing this report (May 11, 2022), 5 (28%) and 6 (43%) patients in the adjuvant and concomitant cohorts, respectively, remained on treatment. Across both cohorts, all ongoing patients have completed 6 cycles of adjuvant trotabresib plus TMZ and are receiving trotabresib monotherapy, including 1 patient in the adjuvant trotabresib 15-mg cohort with an ongoing CR who has been on study treatment for 79 weeks and 1 patient with an ongoing PR in the concomitant trotabresib 30-mg cohort who has been on study treatment for 51 weeks (Figure 5).

## Discussion

Trotabresib, a next-generation BET inhibitor, has demonstrated encouraging antitumor activity as a single agent in preclinical models of glioblastoma and has previously been shown to penetrate the BBB and have durable antitumor activity in patients with high-grade gliomas (V. Moreno, unpublished manuscript).^22^ In this study, trotabresib was well tolerated in combination with TMZ in the adjuvant setting and with TMZ plus RT in the concomitant setting, with a safety profile consistent with studies of trotabresib monotherapy and adjuvant TMZ (V. Moreno, unpublished manuscript).^17,33,34^ Gastrointestinal and hematological TRAEs were the most frequently reported across both cohorts. Nearly all gastrointestinal TRAEs were reported at grade 1/2 severity, with grade 3/4 diarrhea reported in 1 patient who received trotabresib 45 mg in the adjuvant setting and in 1 patient who received trotabresib 30 mg in the concomitant setting. The hematological TRAEs thrombocytopenia and neutropenia were the most frequently reported grade 3/4 TRAEs in both treatment cohorts. Grade 3/4 treatment-related thrombocytopenia was reported in 50% of patients in both cohorts, while grade 3/4 neutropenia was reported in 22% and 29% of patients in the adjuvant and concomitant cohorts, respectively. Most frequently occurring TRAEs were attributed to both study drugs, with the exception of rash and other skin-related TRAEs. A serious TRAE leading to discontinuation of both study drugs (thrombocytopenia) was reported in 1 patient in the adjuvant cohort and was attributed to both trotabresib and TMZ. No other serious TRAEs or TRAEs leading to discontinuation were reported.

At the February 20, 2022, data cutoff, median duration of treatment was 33 weeks (range: 4–72) and 34 weeks (range: 8–54) in the adjuvant and concomitant settings, respectively, which is indicative of promising preliminary efficacy. However, survival data are not yet mature, with median PFS of 7.6 months (95% CI: 3.9–NE) in the adjuvant cohort and NE (95% CI: 5.2 months–NE) in the concomitant cohort. PFS rate at 6 months was 57.8% (95% CI: 31.1–77.3) in the adjuvant setting and 69.2% (95% CI: 37.3–87.2) in the concomitant setting. A previous study has shown that patients treated with SOC TMZ + RT had a median PFS of 6.9 months (95% CI: 5.8–8.2), with a PFS rate at 6 months of 53.9% (95% CI: 48.1–59.6).^3^ However, it is important to note that only 84% of patients in that study had undergone partial or complete resection, with the remaining patients having tumor biopsies only, whereas all patients included in our study had undergone partial or complete resection prior to receiving study treatment. Furthermore, 94% and 79% of patients in the adjuvant and concomitant cohorts of our study, respectively, had wild-type *IDH*, and 50% of patients in the concomitant cohort had methylated *MGMT* promoters, both of which are factors associated with a more favorable prognosis. Taken together, these factors may provide an explanation for at least some of the observed efficacy and highlight the need for caution when comparing results with those of previous studies.

At the time of preparing this report (May 11, 2022), 5 (28%) patients in the adjuvant cohort remained on treatment, including 1 patient at the trotabresib 15-mg dose level with an ongoing CR who has been on study treatment for 79 weeks. In the concomitant cohort, 6 (43%) patients remain on treatment, of whom all have completed adjuvant trotabresib plus TMZ and are receiving trotabresib monotherapy, including 1 patient at the trotabresib 30-mg dose level with an ongoing PR who has been on treatment for 51 weeks.

In this study, trotabresib plasma pharmacokinetics were similar to those observed in clinical trials investigating trotabresib monotherapy, indicating that coadministration with RT and TMZ did not appear to impact trotabresib pharmacokinetics.^17,22,37^ Trotabresib blood pharmacodynamics were consistent with data from studies investigating trotabresib monotherapy.^17,22,37^ In patients receiving trotabresib at the RP2D of 30 mg, blood *CCR1* mRNA expression at 2–4 hours after the fourth dose of trotabresib was ≥50% lower than at baseline in the adjuvant and concomitant settings, which is a reduction that has previously been shown to be associated with response in patients with lymphoma treated with the BET inhibitor CPI-0610.^39^ Blood *HEXIM1* mRNA was increased 2.5- to 4.0-fold following the fourth dose of trotabresib administered at the RP2D in both settings. At 168 hours post-dose in cycle 1, *CCR1* mRNA expression returned to baseline and *HEXIM1* mRNA expression remained above baseline in patients treated with trotabresib at the RP2D in both settings. It is not known when *HEXIM1* mRNA expression returned to baseline because patients did not undergo pharmacodynamic sampling beyond 168 h. However, an exposure–response analysis using pooled data from the current study and 2 other trotabresib studies, found a relationship between time-matched trotabresib plasma concentrations and *HEXIM1* mRNA expression. Blood pharmacodynamics of trotabresib were consistent with its long t_½_ of ~74 hours, which correlates with the observed accumulation of trotabresib in the blood. While *HEXIM1* mRNA expression appeared to be returning towards baseline at 168 hours post-dose, this could be explained by the >75% reduction in plasma trotabresib concentration at this time point, assuming a direct relationship between *HEXIM1* expression and trotabresib exposures.

Blood pharmacodynamic data from the adjuvant cohort showed that the increase in expression of blood *HEXIM1* mRNA was greater between trotabresib doses of 15 and 30 mg than between 30 and 45 mg. Furthermore, data from CC-90010-GBM-001 have demonstrated that trotabresib at a dose of 30 mg results in sufficient exposures to drive BBB penetration, with the study showing measurable concentrations and modulation of pharmacodynamic markers of target engagement in brain tumor tissue.^22^ Importantly, the trotabresib 30 mg 4 days on/24 days off dosing schedule was sufficient to induce BET inhibition, as determined by blood *CCR1* expression, while maintaining platelet nadir >100,000/mm^3^. At a dose of 45 mg, trotabresib may pose a higher risk of thrombocytopenia and have the potential to impact SOC therapy by requiring TMZ dose reductions and/or preventing TMZ dose escalation (Bristol Myers Squibb; data on file). Consequently, the RP2D of trotabresib in combination with TMZ in the adjuvant setting and with TMZ plus RT in the concomitant setting was determined to be 30 mg 4 days on/24 days off.

In conclusion, the RP2D of trotabresib in combination with TMZ in the adjuvant setting and with TMZ plus RT in the concomitant setting was determined to be 30 mg 4 days on/24 days off. Adjuvant trotabresib plus TMZ and concomitant trotabresib plus TMZ and RT appeared to be well tolerated, with encouraging preliminary efficacy supportive of further investigation. The pharmacokinetic profile of trotabresib was consistent with studies investigating trotabresib monotherapy, as were trotabresib pharmacodynamics, which demonstrated target engagement in patients with newly diagnosed glioblastoma. Based on these results, a randomized phase II dose expansion (part B) with a planned enrollment of 162 patients has started, and will compare concomitant trotabresib 30 mg plus TMZ and RT followed by adjuvant trotabresib 30 mg plus TMZ, followed by maintenance with trotabresib 45 mg monotherapy versus SOC therapy.

## Supplementary Material

vdac146_suppl_Supplementary_Material

## Unpublished Papers

V. Moreno. Manuscript submitted to *Neuro-Oncology*.
