# Supplementary material for: Trotabresib (CC-90010) in combination with adjuvant temozolomide or concomitant temozolomide plus radiotherapy in patients with newly diagnosed glioblastoma
Source: Neurooncol Adv. 2022 Oct 28;4(1):vdac146. doi: 10.1093/noajnl/vdac146 (PMC9653173; doi:10.1093/noajnl/vdac146)
Supplement: vdac146_suppl_Supplementary_Material [file vdac146_suppl_supplementary_material.docx]

# Trotabresib (CC‑90010) in combination with adjuvant temozolomide or concomitant temozolomide plus radiotherapy in patients with newly diagnosed glioblastoma

Maria Vieito, Matteo Simonelli, Filip de Vos, Victor Moreno, Marjolein Geurts, Elena Lorenzi, Marina Macchini, Martin J van den Bent, Gianluca Del Conte, Maja de Jonge, Maria Cruz Martín-Soberón, Barbara Amoroso, Tania Sanchez-Perez, Marlene Zuraek, Bishoy Hanna, Ida Aronchik,^1^ Ellen Filvaroff, Henry Chang, Cristina Mendez, Marina Arias Parro, Xin Wei, Zariana Nikolova, Juan Manuel Sepulveda

Vall d’Hebron Institute of Oncology (VHIO), Barcelona, Spain (M.V.); Universidad Autonoma de Barcelona, Barcelona, Spain (M.V.); Department of Biomedical Sciences, Humanitas University, Milan, Italy (M.S.); IRCCS Humanitas Research Hospital, Milan, Italy (M.S., E.L.); Department of Medical Oncology, University Medical Center Utrecht, Utrecht University, Utrecht, the Netherlands (F.D.V.); START Madrid-FJD, Hospital Universitario Fundación Jiménez Díaz, Madrid, Spain (V.M.); Erasmus MC Cancer Institute, Rotterdam, the Netherlands (M.G., M.J.V.D.B., M.D.J.); Department of Oncology, IRCCS San Raffaele Scientific Institute, Milan, Italy (M.M., G.D.C.); Neuro-Oncology Unit, Department of Medical Oncology, Hospital Universitario 12 de Octubre, Madrid, Spain (M.C.M.S.); Centre for Innovation and Translational Research Europe, A Bristol Myers Squibb Company, Seville, Spain (B.A., T.S.P., C.M., Z.N.); Bristol Myers Squibb, San Francisco, CA, USA (M.Z., I.A., E.F., H.C.); Bristol Myers Squibb, Princeton, NJ, USA (B.H., X.W.); Bristol Myers Squibb, Boudry, Switzerland (M.A.P.); Hospital Universitario 12 de Octubre, Madrid, Spain (J.M.S.)

^1^At the time of the study

# Supplementary Material

## Supplementary Figure 1. Trotabresib-mediated tumor growth inhibition in a glioblastoma patient-derived xenograft with and without TMZ.


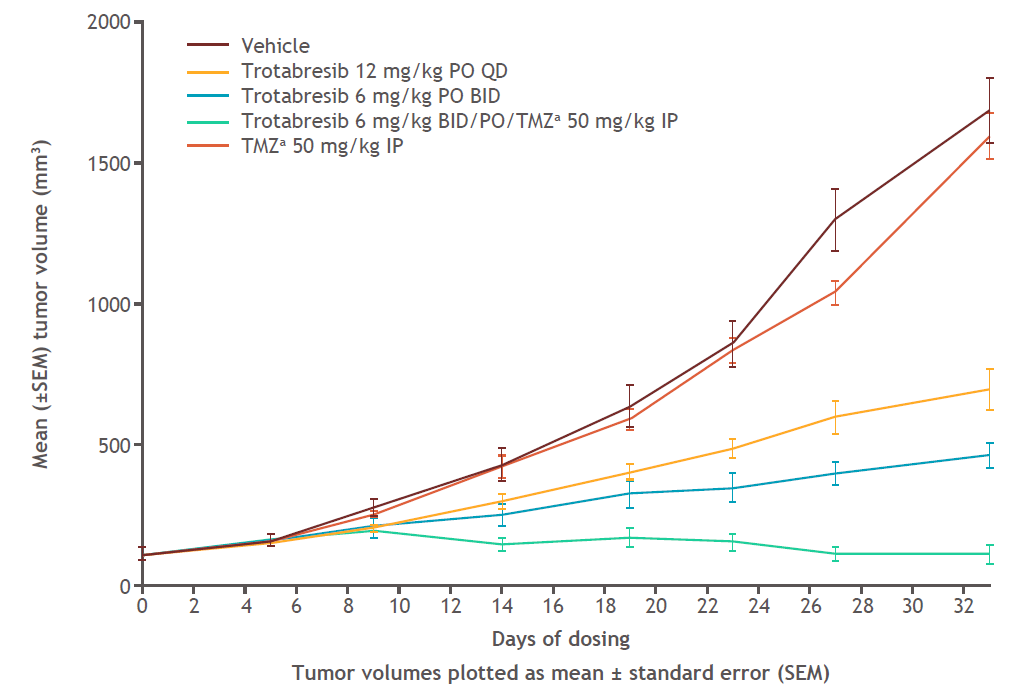
^a^TMZ given on days 7–9 and 22–24.

Abbreviations: BID, twice daily; IP, investigational product; PO, by mouth; QD, once daily; SEM, standard error of the mean; TMZ, temozolomide.

## Supplementary Figure 2. In vivo downregulation of MGMT (A) mRNA by trotabresib as a single agent and (B) protein by trotabresib in combination with TMZ.

**A**


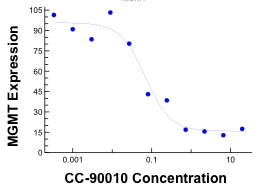


**B**

*
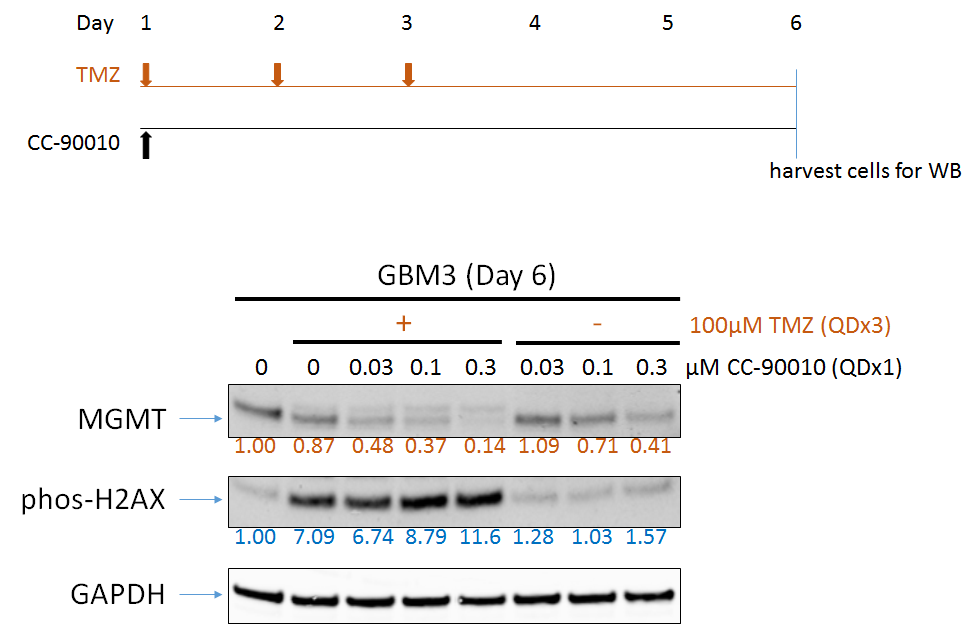
*

TMZ, temozolomide; QD, daily.

## Supplementary Figure 3. Relationship between trotabresib plasma concentration and HEXIM1 mRNA expression.


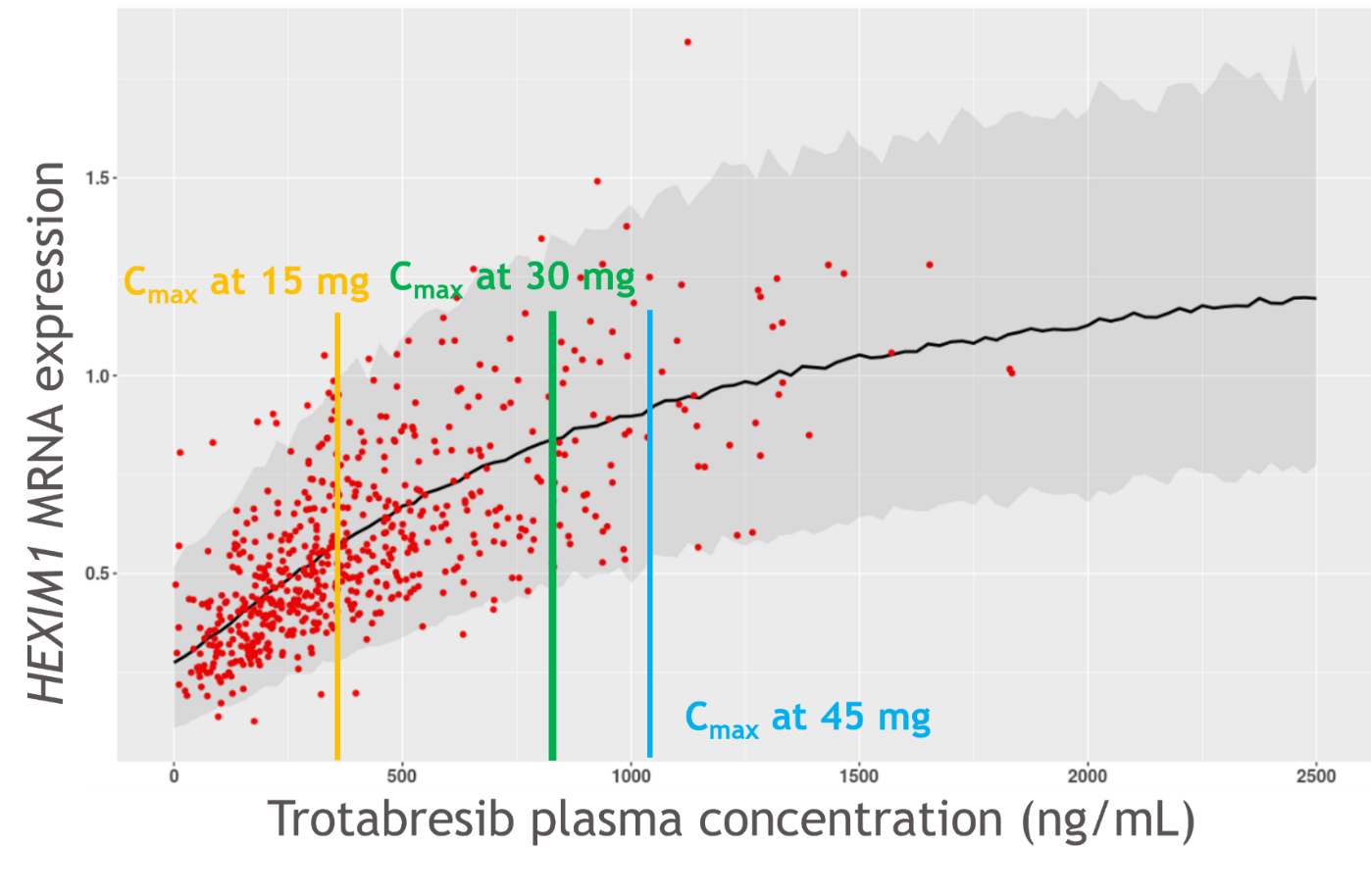


## Supplementary Table 1. Patient Inclusion and Exclusion Criteria

| **Inclusion** | **Exclusion** |
| --- | --- |
| Adults ≥18 years of age | Prior chemotherapy or other anti-tumor treatment for GBM (either approved or investigational) except for surgery and for the Adjuvant Therapy cohort, mandatory concomitant TMZ and RT |
| Newly diagnosed, histologically confirmed WHO grade IV GBM and must have undergone complete or partial resection | Persistent diarrhea due to a malabsorptive syndrome (eg, celiac sprue or inflammatory bowel disease) NCI CTCAE Grade ≥2, despite medical management, or any other significant GI disorder that could affect the absorption of trotabresib |
| Patient must have recovered from the effects of surgery, including post-operative infections or complications   - Toxicities resulting from surgery must have resolved to NCI CTCAE v5.0 Grade ≤1 prior to starting trotabresib treatment (with the exception of grade 2 alopecia) | Symptomatic or uncontrolled ulcers (gastric or duodenal), particularly those with a history of and/or risk of perforation and GI tract hemorrhages |
| For concomitant therapy: prior tumor resection up to 8 weeks prior to first dose of trotabresib | Evidence of recent, symptomatic CNS hemorrhage on baseline MRI or CT scan |
| For adjuvant therapy   - Patient must have recently completed standard or a hypofractionated course of RT with TMZ chemotherapy, and then an MRI documenting SD prior to first dose of trotabresib - All AEs resulting from prior RT with TMZ chemotherapy must have resolved to NCI CTCAE v5.0 Grade ≤1 - Patient must not have experienced significant toxicity to prior RT with TMZ and must have received at least 80% of the planned doses of RT and/or TMZ administered throughout the 42-day concomitant period (up to 49 days) | Requirement of increasing doses of corticosteroids to treat symptomatic cerebral edema within 14 days prior to the first dose of trotabresib |
|  | Known symptomatic acute or chronic pancreatitis |
|  | Impaired cardiac function or clinically significant cardiac diseases |
|  | Pregnant or nursing females |
|  | Known HIV infection |
|  | Known chronic active hepatitis B or C virus infection |
| Life expectancy of at least 3 months | Requirement for ongoing treatment with therapeutic dosing of anticoagulants (eg, warfarin, low molecular weight heparin, Factor Xa inhibitors, thrombin antagonists), or for ongoing prophylactic anticoagulation. Low dose low molecular weight heparin for catheter maintenance was allowed |
| ECOG PS of 0–1 | History of concurrent second cancers requiring active and ongoing systemic treatment, except non-melanoma skin cancer, completely resected cervical carcinoma in situ, low risk prostate cancer (cT1-2a N0 and Gleason score ≤6 and PSA <10 ng/mL), either totally resected or irradiated with curative intent (with PSA ≤0.1 ng/mL) or under active surveillance. Other cancers for which the subject has completed potentially curative treatment more than 3 years prior to study entry are allowed |
| The following laboratory values at screening:   - Absolute neutrophil count ≥ 1.5 × 10^9^/L without growth factor support for 7 days (14 days if patient received pegfilgrastim) - Hemoglobin ≥10 g/dL - Platelet count ≥150 × 10^9^/L - Serum potassium concentration within normal range, or correctable with supplements - Serum glutamic oxaloacetic transaminase/aspartate aminotransferase and serum glutamate pyruvic transaminase/alanine aminotransferase ≤3.0 × ULN - Serum total bilirubin ≤1.5 × ULN - Serum creatinine ≤1.5 × ULN or measured glomerular filtration rate ≥50 mL/min/1.73 m^2^ using an exogenous filtration marker such as iohexol, inulin, 51Cr EDTA or 1^125^ iothalamate, or creatinine clearance of ≥50 mL/min using Cockcroft-Gault equation - Serum albumin >3.5 g/dL - PT (or INR) and APTT within normal range | Evidence of history of bleeding diathesis. Any hemorrhage/bleeding event > CTCAE grade 2 or hemoptysis >1 teaspoon within 4 weeks prior to the first dose of trotabresib |
|  | Known prior episodes of non-arteritic anterior ischemic optic neuropathy |
|  | Any significant medical condition (eg, active or uncontrolled infection, hepatic or renal disease), laboratory abnormality, or psychiatric illness that would prevent the patient from participating (or compromise compliance) in the study or would place the patient at unacceptable risk if he/she were to participate in the study |
|  | Patient with any condition that confounds the ability to interpret data from the study |
|  | Patient with poor bone marrow reserve as assessed by Investigator such as in conditions requiring regular hematopoietic support (blood or platelet transfusions, erythropoietin, granulocyte colony stimulating factor or other hematopoietic growth factors) |

Abbreviations: AE, adverse event; APTT, activated partial thromboplastin time; CNS, central nervous system; CT, computed tomography; CTCAE, Common Terminology Criteria for Adverse Events; ECOG PS, Eastern Cooperative Oncology Group performance status; EDTA, ethylenediaminetetraacetic acid; GBM, glioblastoma; GI, gastrointestinal; HIV, human immunodeficiency virus; INR, international normalized ratio; MRI, magnetic resonance imaging; NCI, National Cancer Institute; PSA, prostate-specific antigen; PT, prothrombin time; RT, radiotherapy; SD, stable disease; TMZ, temozolomide; ULN, upper limit of normal; WHO, World Health Organization.

## Supplementary Table 2. DLT Definitions

| **Definition** |
| --- |
| Hyperglycemia meeting any of the following criteria:   - Grade 2 fasting hyperglycemia (>160 mg/dL) lasting >14 days despite optimal medical treatment - Grade 3 or greater fasting hyperglycemia (>250 mg/dL) lasting >4 days despite optimal medical treatment - Grade 4 hyperglycemia lasting ≥12 hours despite adequate treatment - Hyperglycemia associated with diabetic ketoacidosis or nonketotic hyperosmolar coma regardless of glucose level - Hyperglycemia that necessitates dose reduction despite dose interruption and resolution to ≤ grade 1 hyperglycemia within two weeks (fasting glucose is defined as a level monitored ≥4 hours from the last meal for assessment of DLT) |
| Any grade 4 non-hematologic toxicity of any duration |
| Any non-hematologic toxicity grade ≥3 EXCEPT for:   - Grade 3 diarrhea, nausea, or vomiting of ≤3 days duration (with optimal medical management) - Grade 3 rash of the acneiform, pustular, or maculopapular type which resolves to grade ≤2 within 7 days of study‑drug interruption and does not recur at the same level with resumption of study drug at the same dose (with optimal medical management) - Grade 3 fatigue which resolves to grade ≤2 within 7 days of study drug interruption and does not recur at the same level with resumption of study drug at the same dose (with optimal medical management) |
| Hematological toxicities as follows:   - Febrile neutropenia - Grade 4 neutropenia lasting >7 days - Grade 4 thrombocytopenia lasting >7 days - Grade ≥3 thrombocytopenia with clinically significant bleeding |
| Any AE, grade ≥2 not specified or exempt above, unless clearly determined to be unrelated to the drug, necessitating dose-level reduction during cycle 1 |
| Isolated laboratory changes without associated clinical signs or symptoms (eg, hypomagnesemia, hypermagnesemia, hypoalbuminemia, hypophosphatemia, and lymphocyte count increased or decreased) may not be included in this definition |

Abbreviations: AE, adverse; DLT, dose‑limiting toxicity.

## Supplementary Table 3. TRAEs reported in ≥ 2 patients at any grade or in ≥1 patient at grade 3/4 severity in the overall adjuvant or concomitant cohorts by relationship to study drug

|  | **Adjuvant Trotabresib + TMZ Overall (N = 18)** | | | | | | **Concomitant Trotabresib + TMZ + RT Overall (N = 14)** | | | | | |
| --- | --- | --- | --- | --- | --- | --- | --- | --- | --- | --- | --- | --- |
|  | **Any study drug** | | **Trotabresib** | | **TMZ** | | **Any study drug** | | **Trotabresib** | | **TMZ** | |
| **TRAEs, n (%)** | **Any grade** | **Grade 3/4** | **Any grade** | **Grade 3/4** | **Any grade** | **Grade 3/4** | **Any grade** | **Grade 3/4** | **Any grade** | **Grade 3/4** | **Any grade** | **Grade 3/4** |
| **Any TRAE** | 17 (94) | 10 (56) | 16 (89) | 9 (50) | 17 (94) | 10 (56) | 14 (100) | 9 (64) | 14 (100) | 7 (50) | 14 (100) | 9 (64) |
| Nausea | 12 (67) | 0 | 9 (50) | 0 | 12 (67) | 0 | 11 (79) | 0 | 8 (57) | 0 | 11 (79) | 0 |
| Thrombocytopenia | 13 (72) | 9 (50) | 10 (56) | 7 (39) | 13 (72) | 9 (50) | 10 (71) | 7 (50) | 9 (64) | 6 (43) | 10 (71) | 7 (50) |
| Vomiting | 7 (39) | 0 | 4 (22) | 0 | 7 (39) | 0 | 8 (57) | 0 | 5 (36) | 0 | 8 (57) | 0 |
| Dysgeusia | 8 (44) | 0 | 6 (33) | 0 | 4 (22) | 0 | 6 (43) | 0 | 5 (36) | 0 | 4 (29) | 0 |
| Diarrhea | 11 (61) | 1 (6) | 11 (61) | 1 (6) | 5 (28) | 0 | 3 (21) | 1 (7) | 2 (14) | 1 (7) | 3 (21) | 0 |
| Decreased appetite | 8 (44) | 0 | 6 (33) | 0 | 6 (33) | 0 | 5 (36) | 0 | 3 (21) | 0 | 4 (29) | 0 |
| Asthenia | 9 (50) | 1 (6) | 7 (39) | 1 (6) | 6 (33) | 1 (6) | 4 (29) | 0 | 3 (21) | 0 | 4 (29) | 0 |
| Neutropenia | 7 (39) | 4 (22) | 5 (28) | 3 (17) | 7 (39) | 4 (22) | 6 (43) | 4 (29) | 6 (43) | 4 (29) | 6 (43) | 4 (29) |
| Hyperbilirubinemia | 7 (39) | 1 (6) | 6 (33) | 1 (6) | 3 (17) | 0 | 2 (14) | 0 | 2 (14) | 0 | 2 (14) | 0 |
| Anemia | 4 (22) | 0 | 4 (22) | 0 | 3 (17) | 0 | 5 (36) | 2 (14) | 3 (21) | 2 (14) | 4 (29) | 2 (14) |
| Fatigue | 2 (11) | 0 | 2 (11) | 0 | 2 (11) | 0 | 5 (36) | 1 (7) | 5 (36) | 1 (7) | 5 (36) | 1 (7) |
| Rash | 5 (28) | 0 | 5 (28) | 0 | 1 (6) | 0 | 1 (7) | 0 | 1 (7) | 0 | 0 | 0 |
| Acneiform dermatitis | 3 (17) | 0 | 3 (17) | 0 | 1 (6) | 0 | 3 (21) | 0 | 3 (21) | 0 | 2 (14) | 0 |
| Dyspepsia | 2 (11) | 0 | 2 (11) | 0 | 2 (11) | 0 | 3 (21) | 0 | 2 (14) | 0 | 2 (14) | 0 |
| Hyperglycemia | 4 (22) | 1 (6) | 4 (22) | 1 (6) | 1 (6) | 0 | 1 (7) | 0 | 1 (7) | 0 | 0 | 0 |
| Lymphopenia | 2 (11) | 1 (6) | 2 (11) | 1 (6) | 2 (11) | 1 (6) | 3 (21) | 1 (7) | 2 (14) | 0 | 3 (21) | 1 (7) |
| Pruritus | 3 (17) | 0 | 3 (17) | 0 | 0 | 0 | 0 | 0 | 0 | 0 | 0 | 0 |
| Leukopenia | 2 (11) | 0 | 1 (6) | 0 | 2 (11) | 0 | 1 (7) | 0 | 0 | 0 | 1 (7) | 0 |
| AST increased | 2 (11) | 0 | 1 (6) | 0 | 2 (11) | 0 | 1 (7) | 0 | 1 (7) | 0 | 1 (7) | 0 |
| Hypophosphatemia | 2 (11) | 0 | 2 (11) | 0 | 1 (6) | 0 | 1 (7) | 0 | 1 (7) | 0 | 1 (7) | 0 |
| ALT increased | 2 (11) | 1 (6) | 1 (6) | 0 | 2 (11) | 1 (6) | 1 (7) | 1 (7) | 1 (7) | 1 (7) | 1 (7) | 1 (7) |
| Hypotension | 2 (11) | 0 | 2 (11) | 0 | 0 | 0 | 0 | 0 | 0 | 0 | 0 | 0 |
| Insomnia | 2 (11) | 0 | 2 (11) | 0 | 1 (6) | 0 | 0 | 0 | 0 | 0 | 0 | 0 |
| Seizure | 0 | 0 | 0 | 0 | 0 | 0 | 2 (14) | 0 | 2 (14) | 0 | 0 | 0 |
| Blood bilirubin increased | 0 | 0 | 0 | 0 | 0 | 0 | 2 (14) | 0 | 2 (14) | 0 | 1 (7) | 0 |
| Intracranial hemorrhage | 1 (6) | 1 (6) | 1 (6) | 1 (6) | 0 | 0 | 0 | 0 | 0 | 0 | 0 | 0 |

Abbreviations: ALT, alanine aminotransferase; AST, aspartate aminotransferase; RT, radiotherapy; TMZ, temozolomide; TRAE, treatment-related adverse event.

## Supplementary Table 4. TRAEs related to any study drug reported in ≥2 patients at any grade or in ≥1 patient at grade 3/4 severity in the overall adjuvant or concomitant cohorts by trotabresib dose level

|  | **Adjuvant Trotabresib + TMZ** | | | | | | **Concomitant Trotabresib + TMZ + RT** | | | |
| --- | --- | --- | --- | --- | --- | --- | --- | --- | --- | --- |
|  | **15 mg (n = 5)** | | **30 mg (n = 6)** | | **45 mg (n = 7)** | | **15 mg (n = 6)** | | **30 mg (n = 8)** | |
| **TRAEs, n (%)** | **Any grade** | **Grade 3/4** | **Any grade** | **Grade 3/4** | **Any grade** | **Grade 3/4** | **Any grade** | **Grade 3/4** | **Any grade** | **Grade 3/4** |
| **Any TRAE** | 4 (80) | 2 (40) | 6 (100) | 4 (67) | 7 (100) | 4 (57) | 6 (100) | 3 (50) | 8 (100) | 6 (75) |
| Nausea | 3 (60) | 0 | 5 (83) | 0 | 4 (57) | 0 | 5 (83) | 0 | 6 (75) | 0 |
| Vomiting | 2 (40) | 0 | 2 (33) | 0 | 3 (43) | 0 | 3 (50) | 0 | 5 (63) | 0 |
| Diarrhea | 2 (40) | 0 | 3 (50) | 0 | 6 (86) | 1 (14) | 1 (17) | 0 | 2 (25) | 1 (13) |
| Dyspepsia | 0 | 0 | 2 (33) | 0 | 0 | 0 | 0 | 0 | 3 (38) | 0 |
| Thrombocytopenia | 3 (60) | 2 (40) | 5 (83) | 4 (67) | 5 (71) | 3 (43) | 4 (67) | 2 (33) | 6 (75) | 5 (63) |
| Neutropenia | 1 (20) | 0 | 5 (83) | 4 (67) | 1 (14) | 0 | 2 (33) | 0 | 4 (50) | 4 (50) |
| Anemia | 1 (20) | 0 | 2 (33) | 0 | 1 (14) | 0 | 2 (33) | 1 (17) | 3 (38) | 1 (13) |
| Lymphopenia | 0 | 0 | 2 (33) | 1 (17) | 0 | 0 | 1 (17) | 1 (17) | 2 (25) | 0 |
| Asthenia | 3 (60) | 0 | 3 (50) | 1 (17) | 3 (43) | 0 | 2 (33) | 0 | 2 (25) | 0 |
| Fatigue | 0 | 0 | 1 (17) | 0 | 1 (14) | 0 | 3 (50) | 0 | 2 (25) | 1 (13) |
| Decreased appetite | 2 (40) | 0 | 3 (50) | 0 | 3 (43) | 0 | 2 (33) | 0 | 3 (38) | 0 |
| Hyperglycemia | 0 | 0 | 1 (17) | 0 | 3 (43) | 1 (14) | 1 (17) | 0 | 0 | 0 |
| Dermatitis acneiform | 0 | 0 | 2 (33) | 0 | 1 (14) | 0 | 2 (33) | 0 | 1 (13) | 0 |
| Rash | 3 (60) | 0 | 2 (33) | 0 | 0 | 0 | 0 | 0 | 1 (13) | 0 |
| Pruritus | 1 (20) | 0 | 2 (33) | 0 | 0 | 0 | 0 | 0 | 0 | 0 |
| Dysgeusia | 2 (40) | 0 | 3 (50) | 0 | 3 (43) | 0 | 4 (67) | 0 | 2 (25) | 0 |
| Seizure | 0 | 0 | 0 | 0 | 0 | 0 | 2 (33) | 0 | 0 | 0 |
| Hyperbilirubinemia | 1 (20) | 0 | 2 (33) | 1 (17) | 4 (57) | 0 | 1 (17) | 0 | 1 (13) | 0 |
| ALT increased | 0 | 0 | 1 (17) | 0 | 1 (14) | 1 (14) | 0 | 0 | 1 (13) | 1 (13) |
| Blood bilirubin increased | 0 | 0 | 0 | 0 | 0 | 0 | 0 | 0 | 2 (25) | 0 |
| Hemorrhage intracranial | 0 | 0 | 1 (17) | 1 (17) | 0 | 0 | 0 | 0 | 0 | 0 |
| Leukopenia | 1 (20) | 0 | 1 (17) | 0 | 0 | 0 | 0 | 0 | 1 (13) | 0 |
| Hypophosphatemia | 0 | 0 | 1 (17) | 0 | 1 (14) | 0 | 1 (17) | 0 | 0 | 0 |
| AST increased | 0 | 0 | 1 (17) | 0 | 1 (14) | 0 | 0 | 0 | 1 (13) | 0 |
| Insomnia | 1 (20) | 0 | 0 | 0 | 1 (14) | 0 | 0 | 0 | 0 | 0 |
| Hypotension | 1 (20) | 0 | 0 | 0 | 1 (14) | 0 | 0 | 0 | 0 | 0 |

Abbreviations: ALT, alanine aminotransferase; AST, aspartate aminotransferase; RT, radiotherapy; TMZ, temozolomide; TRAE, treatment-related adverse event.

## Supplementary Table 5. Patients who discontinued treatment due to adverse events at the February 20, 2022, data cutoff

|  | **Adjuvant Trotabresib + TMZ** | | | | **Concomitant Trotabresib + TMZ + RT** | | |
| --- | --- | --- | --- | --- | --- | --- | --- |
|  | **15 mg**  **(n = 5)** | **30 mg**  **(n = 6)** | **45 mg**  **(n = 7)** | **Overall**  **(N = 18)** | **15 mg**  **(n = 6)** | **30 mg**  **(n = 8)** | **Overall**  **(N = 14)** |
| **TEAEs leading to discontinuation of any study drug** | | | | | | | |
| Number of patients, n (%)  Trotabresib or TMZ  Trotabresib  TMZ | 0  0  0 | 1 (17)  1 (17)  1 (17) | 1 (14)  1 (14)  1 (14) | 2 (11)  2 (11)  2 (11) | 0  0  0 | 0  0  0 | 0  0  0 |
| **TRAEs leading to discontinuation of any study drug** | | | | | | | |
| Number of patients, n (%)  Related to any study drug  Related to trotabresib  Related to TMZ | 0  0  0 | 1 (17)  1 (17)  1 (17) | 0  0  0 | 1 (6)  1 (6)  1 (6) | 0  0  0 | 0  0  0 | 0  0  0 |
| **TRAEs leading to discontinuation of trotabresib** | | | | | | | |
| Number of patients, n (%)  Related to any study drug  Related to trotabresib  Related to TMZ | 0  0  0 | 1 (17)  1 (17)  1 (17) | 0  0  0 | 1 (6)  1 (6)  1 (6) | 0  0  0 | 0  0  0 | 0  0  0 |
| **TRAEs leading to discontinuation of TMZ** | | | | | | | |
| Number of patients, n (%)  Related to any study drug  Related to trotabresib  Related to TMZ | 0  0  0 | 1 (17)  1 (17)  1 (17) | 0  0  0 | 1 (6)  1 (6)  1 (6) | 0  0  0 | 0  0  0 | 0  0  0 |

Abbreviations: RT, radiotherapy; TEAE, treatment-emergent adverse event; TMZ, temozolomide; TRAE, treatment-related adverse event.

## Supplementary Table 6. Treatment duration and relative dose intensities^a^ for trotabresib and TMZ at the February 20, 2022, data cutoff

|  | **Adjuvant Trotabresib + TMZ** | | | | **Concomitant Trotabresib + TMZ + RT** | | |
| --- | --- | --- | --- | --- | --- | --- | --- |
| **Dose level** | 15 mg | 30 mg | 45 mg | Overall | 15 mg | 30 mg | Overall |
| **Trotabresib concomitant stage, n** | – | – | – | – | 6 | 8 | 14 |
| Median treatment duration (range), weeks | – | – | – | – | 8.00  (8.0–8.1) | 8.00  (7.9–8.0) | 8.00  (7.9–8.1) |
| Median relative dose intensity (range), % | – | – | – | – | 100.00  (98.2–100.0) | 100.00  (75.0–101.8) | 100.00  (75.0–101.8) |
| **Trotabresib adjuvant stage, n** | 5 | 6 | 7 | 18 | 6 | 7 | 13 |
| Median treatment duration (range), weeks | 23.86  (19.1–26.3) | 26.07  (4.1–28.3) | 24.00  (4.1–33.0) | 24.00  (4.1–33.0) | 24.00  (0.7–26.6) | 22.14  (13.0–26.1) | 23.57  (0.7–26.6) |
| Median relative dose intensity (range), % | 94.60  (83.6–100.6) | 92.17  (58.0–98.2) | 96.55  (56.6–100.0) | 95.03  (56.6–100.6) | 99.26  (82.8–560.0) | 92.31  (21.5–108.4) | 94.92  (21.5–560.0) |
| **Trotabresib monotherapy stage, n** | 3 | 4 | 4 | 11 | 4 | 2 | 6 |
| Median treatment duration (range), weeks | 24.71  (7.0–48.0) | 12.50  (4.0–36.1) | 10.00  (0.4–28.0) | 12.00  (0.4–48.0) | 15.14  (7.6–19.3) | 3.79  (3.6–4.0) | 10.79  (3.6–19.3) |
| Median relative dose intensity (range), % | 90.28  (83.6–114.3) | 93.14  (62.7–100.0) | 100.00  (100.0–700.0) | 100.00  (62.7–700.0) | 77.41  (66.0–98.2) | 64.00  (28.0–100.0) | 77.41  (28.0–100.0) |
| **TMZ concomitant stage, n** | – | – | – | – | 6 | 8 | 14 |
| Median treatment duration (range), weeks | – | – | – | – | 6.00  (5.3–6.1) | 6.00  (5.7–6.1) | 6.00  (5.3–6.1) |
| Median relative dose intensity (range), % | – | – | – | – | 100.00  (81.0–100.0) | 96.46  (83.3–100.0) | 98.84  (81.0–100.0) |
| **TMZ adjuvant stage, n** | 5 | 6 | 7 | 18 | 6 | 7 | 13 |
| Median treatment duration (range), weeks | 23.86  (19.1–26.3) | 26.07  (4.1–28.3) | 24.00  (4.1–33.0) | 24.00  (4.1–33.0) | 24.00  (0.7–26.6) | 22.14  (13.0–26.1) | 23.57  (0.7–26.6) |
| Median relative dose intensity (range), % | 89.87  (72.3–96.4) | 71.39  (60.1–91.5) | 75.00  (39.4–95.8) | 76.68  (39.4–96.4) | 89.28  (53.8–420.0) | 80.61  (55.4–108.4) | 80.77  (53.8–420.0) |

^a^Defined as the actual dose intensity/protocol-expected dose intensity.
Abbreviations: RT, radiotherapy; TMZ, temozolomide.

## Supplementary Table 7. Summary of PFS at the February 20, 2022, data cutoff

|  | **Adjuvant Trotabresib + TMZ** | | | | **Concomitant Trotabresib + TMZ + RT** | | |
| --- | --- | --- | --- | --- | --- | --- | --- |
| **Dose level** | **15 mg  (n = 5)** | **30 mg  (n = 6)** | **45 mg  (n = 7)** | **Overall  (n = 18)** | **15 mg  (n = 6)** | **30 mg  (n = 8)** | **Overall  (n = 14)** |
| **Median PFS, months (95% CI)** | 7.7 (5.0–NE) | 6.9 (1.7–NE) | 7.6 (3.9–NE) | 7.6 (3.9–NE) | NE (2.1–NE) | 7.2 (2.4–NE) | NE (5.2–NE) |
| Range | 4.96–15.52 | 1.68–12.03 | 0.03–11.08 | 0.03–15.52 | 2.10–10.68 | 0.03–7.92 | 0.03–10.68 |
| **6-month PFS rate, % (95% CI)** | 60.0 (12.6–88.2) | 50.0 (11.1–80.4) | 66.7 (19.5–90.4) | 57.8 (31.1–77.3) | 66.7 (19.5–90.4) | 71.4 (25.8–92.0) | 69.2 (37.3–87.2) |

Abbreviations: CI, confidence interval; NE, not estimable; PFS, progression-free survival; RT, radiotherapy; TMZ, temozolomide.

## Supplementary Table 8. BOR at the February 20, 2022, data cutoff

|  | **Adjuvant Trotabresib + TMZ** | | | | **Concomitant Trotabresib + TMZ + RT** | | |
| --- | --- | --- | --- | --- | --- | --- | --- |
| **Dose level** | **15 mg  (n = 5)** | **30 mg  (n = 6)** | **45 mg  (n = 7)** | **Overall  (n = 18)** | **15 mg  (n = 6)** | **30 mg  (n = 8)** | **Overall  (n = 14)** |
| **BOR, n (%)**  CR  PR  SD  PD  NE | 1 (20)  0  4 (80)  0  0 | 0  0  4 (67)  2 (33)  0 | 0  0  6 (86)  0  1 (14) | 1 (6)  0  14 (78)  2 (11)  1 (6) | 0  0  5 (83)  1 (17)  0 | 0  1 (13)  6 (75)  0  1 (13) | 0  1 (7)  11 (79)  1 (7)  1 (7) |

Abbreviations: BOR, best overall response; CR, complete response; NE, not evaluable; PD, progressive disease; PR, partial response; RT, radiotherapy; SD, stable disease; TMZ, temozolomide.
